# Spatial spillover effects of capital factor agglomeration on the urban industrial structure upgrading in China: Based on panel data of 284 prefecture-level cities

**DOI:** 10.1371/journal.pone.0258758

**Published:** 2021-10-19

**Authors:** Maosheng Ran, Cheng Zhao

**Affiliations:** School of Economics and Business Administration, Chongqing University, Chongqing, China; Northeastern University (Shenyang China), CHINA

## Abstract

The spatial agglomeration of capital factors has become an important force affecting regional economic development and industrial structure. Investigating the spatial relationship of capital factor agglomeration is a key way to accelerate the upgrading of urban industrial structure and realize sustainable development. Based on the panel data of 284 cities in China from 2008 to 2017, we use the theoretical framework of spatial econometrics and estimate the spatial effects of capital factor agglomeration on the upgrading of urban industrial structure. Both the global Moran index and the local Moran scatter chart present that the agglomeration of capital factors and the upgrading index of urban industrial structure shows the characteristics of spatial agglomeration. The results reveal that the agglomeration of capital factors can significantly promote the upgrading of the industrial structure of local and surrounding cities. Still, the spatial spillover effect is not significant. We then explore the possible factors that limit the spatial spillover effects of capital agglomeration. Using the results of the paper, we provide policy suggestions for strengthening urban industrial construction and optimizing the urban governance model.

## Introduction

China’s economic development has entered a new normal, gradually changing from a stage of rapid growth to a stage of high-quality development, and it is currently in a critical period of changing the mode of development and the driving force of its growth in order to optimize economic structure. Promoting the upgrading of industrial structure is of great strategic significance in enhancing China’s economic innovation and competitiveness. The capital factors are the basic elements of economic development and the carriers of labor and technology, as well as other dynamics. The agglomeration of capital factors is a unique means of resource allocation in modern economic. It is the embodiment of optimal allocation of capital factors for the entire economy. As the core driving force of urban economic development, capital factors will also be affected by the role of urban space economic development and transformation in China. Because of the differences in location factors, natural endowments, human and social environments, and economic development, capital factors show agglomeration characteristics within specific areas. There is a relative lack of research concerning capital factors and the upgrading of urban industrial structure from the perspective of spatial agglomeration. Therefore, it is of theoretical and practical significance to study the impact of the spatial agglomeration of capital factors on the upgrading of industrial structure.

Within the theory of economic growth, as one of the two most basic factors of production function, capital factors are of utmost significance to regional economic growth [1]. The agglomeration of capital factors is not a direct accumulation of factors, but rather an integration among factors formed by geographic concentration, which can realize cross-regional flow and allocation, and produce polarization effects and trickle-down effects in the urban economy [2]. Tappeiner found an obvious spatial correlation in the input of regional capital factors [3]. The relationship between capital factors and economic growth has received much attention from academic circles. Still, few studies have noticed that the impact of capital factors on economic growth is mainly achieved through the adjustment of industrial structure. Capital factor agglomeration greatly affects the speed and efficiency of industrial structure upgrading.

On the one hand, capital factor agglomeration reduces transaction costs through economies of scope and economies of scale, thus alleviating the financial constraints of industrial structure upgrading [4]. On the other hand, capital is profit-oriented and will prioritize flowing into industries with higher potential profit margins. It will accelerate the transfer of other factors of production from inefficient industries to high-efficiency industries, and the resulting structural dividend will maintain sustained economic growth [5,6]. Empirical research shows that American capital investment plays a very obvious role in promoting production efficiency [7]. In the study of the spillover effect of factor agglomeration, Zhang holds that there should be a complementary match between the agglomeration factor and the local factor structure to give full play to the local absorptive capacity and to maximize the spillover effect of the agglomeration factor [8].

In the research regarding the influence of capital factors, some studies have concluded that the function of the capital market is mostly realized through agglomeration of capital factors [9–11]. For example, Wang studied the spatial spillover impact of the capital market on industrial structure upgrading, and believes that direct financing has the most significant impact on regional industrial upgrading [12]. Wu studied the spatial effect of venture capital agglomeration on regional innovation capability [13]. Park & Musa found that in areas with a high level of capital factors agglomeration, this dynamic is conducive to information exchange and resource sharing, which can make more effective use of existing network systems and infrastructure to achieve economies of scale [14]. Wurgler uses the data regarding manufacturing output value and the total investment of 65 countries to confirm that the more developed the capital market is, the higher the efficiency of resource allocation is. The study shows that the agglomeration of capital factors is beneficial in improving the efficiency of resource allocation and promoting the upgrading of industrial structure [15]. Places with a high level of capital factors agglomeration usually accumulate more innovation resources, so the speed of product upgrading and innovation in such a region is higher than that in other regions. The agglomeration of capital factors can provide financial support and long-term incentives for venture capitalists, thus promoting the long-term stabilization of technological innovation behavior [16]. However, few studies give attention to the spatial correlation of the agglomeration of capital factors. In fact, the role and function of capital agglomeration can not only serve the goal of upgrading local industrial structure, but can also affect the transformation of the industrial structure in the surrounding areas. If the spatial spillover effect is ignored, the research results show a certain deviation.

The development of information and communication technology has allowed funds to overcome the limitations of geographical space and realize long-distance, low-cost connections and transactions [17]. Some scholars adhere to the viewpoint of "the end of geography" [18]. However, Henry Chen’s research shows that geographical location is closely related to the results of venture capital [19]. Some scholars also believe that the information needed for urban industrial upgrading will result in of information asymmetry with the increase of geographical distance, thus affecting the spillover effect of capital factors on the upgrading of urban industrial structure [20]. The spatial effect of capital factor agglomeration also has a specific regional boundary [21]. Yu believes that local protectionism will result in the spatial spillover effect of capital agglomeration having certain regional boundaries, which leads to the spatial spillover effect being restricted by geographical distance [22].

The development of the urban industrial structure will be affected by the local and surrounding cities. The first law of geography states that everything is related to other things, and the closer the distance between two things, the stronger the relevance of that relationship is. In empirical research, the spatial econometric model is widely used to study spatial spillover [23–25]. For example, Zheng believes that the human capital spillover effect and the market integration effect strengthen China’s urban agglomeration [26]. Yu used a spatial econometric model to study the impact of the spatial agglomeration of foreign direct investment on urban green total factor productivity in China [27]. However, from the perspective of agglomeration, there are few studies on the spatial distribution characteristics and spatial spillover effects of capital agglomeration on upgrading of urban industrial structure. The agglomeration of the economy in geographical space has become a significant feature of industrialization [28], raising the question of whether there is a spatial difference and spatial spillover effect of capital agglomeration on upgrading the urban industrial structure.

Compared with previous studies, the contribution of this paper is mainly focused in three areas. First, this paper brings the agglomeration of capital factors and the upgrading of urban industrial structure into the same analytical framework. It uses the geographical distance between cities as a spatial weight matrix to replace the adjacency matrix and lead to a more accurate research conclusion. Second, previous studies paid less attention to the spatial correlation of capital factor agglomeration. Based on a full consideration of this spatial correlation, this paper uses the spatial panel econometric model to empirically test the spatial transmission mechanism of capital factor agglomeration and measure the spatial spillover effect. Third, regarding data selection and using the statistical data of 284 prefecture-level cities in China from 2008 to 2017, this paper analyzes the spatial spillover effect of capital factor agglomeration on upgrading industrial structure. Compared with provincial data, the research conclusions based on urban data are of more practical significance.

The rest of the paper proceeds as follows. Section 2 discusses the the model and data. The regression results and consequent discussion are presented in Section 3. Conclusions and policy implications are presented in Section 4.

## Model setting and data

### Model setting

When studying the problems of industrial upgrading and regional development, the previous literature often assumes that regional variables are independent. However, according to the first geographical law of Toble, the spatial effects of different regions should be considered when studying the development of the regional industry. To verify the spatial spillover effect of capital factor agglomeration and urban industrial structure upgrading, this paper establishes the following spatial econometric model.

$$I_{it}=\rho \sum_{i=1}^{N}W_{ij}\mathrm{Iit}+\beta \;\ln kclu_{it}+\lambda \sum_{i=1}^{N}W_{ij}\ln kclu_{it}+\delta X_{it}+\alpha_{i}+\gamma t+\varepsilon_{it}$$ (1)

where *I*_*it*_ represents the upgrading degree of the industrial structure of city *i* in year *t*, ρ is the spatial lag coefficient, β represents the spatial spillover coefficient of capital factor agglomeration, X is the control variable, *W*_*ij*_ represents the geospatial weight matrix, and the linear distance between cities is used as the weight. *α*_*i*_ and γ_t_ represent the regional effect and time effect, respectively. *ε*_*it*_ is a random disturbance term.

### Variable description

#### The level of industrial upgrading structure

The main explained variable we explore in this paper concerns the upgrading level of industrial structure, which is mainly measured from two dimensions: the optimization of industrial structure and the rationalization of industrial structure. The optimization of industrial structure is an important dimension in industrial structure upgrading, reflecting the dynamic process of the sequential evolution of industrial structure from a low-level state to a high-level state. This method refers to Yuan [29]. The hierarchical coefficient of industrial structure expresses the industrial Industrial structure optimization (ais), that is, the relative change in the proportion of share. The specific calculation formula is as follows:

$$ais=\sum_{m=1}^{3}y_{i,m,t}*m,m=1,2,3$$ (2)

where y_*i*,*m*,*t*_ denotes the proportion of industry m in the GDP of region i in period t.

The rationalization of industrial structure refers to the aggregate quality among industries. On the one hand, it should reflect the degree of coordination between industries, on the other hand, it should also reflect the degree of effective utilization of resources. Thus, it is a measure of the coupling degree between factor input and output structures. The theory of resource allocation, which is the predominate theory in evaluating the rationalization of industrial structures, considers the importance of the allocation, coordination and utilization efficiency of factor resources among industries [30].

Scholars generally use the Theil index to study regional inequality and income inequality [31]. In this paper, we refer to the method of Gan et al [30] and use the Theil index to measure the rationalization of industrial structure in prefecture-level cities. It can measure the structural deviation of output value, employment and economic status in different industries and reflect the structure of output value and employment in China’s three major industrial sectors. The specific calculation formula is as follows:

$$theil=\sum_{m=1}^{3}y_{i,m,t}\;\ln\;\left(y_{i,m,t}/l_{i,m,t}\right),\;m=1,2,3$$ (3)

where y_*i*,*m*,*t*_ denotes the proportion of industry m in the GDP of region i in period t. l_*i*,*m*,*t*_ indicates the proportion of employees in industry *m* of region *i* during period *t*. The higher its value is, the greater the economy deviates from the equilibrium state and the more unreasonable the industrial structure is.

#### The level of capital factors agglomeration

The index of location entropy can measure the level of industrial agglomeration in a region. It can truly reflect the spatial distribution of factors in a city while eliminating the difference of regional scale. In this paper, the location entropy index is selected to measure each city’s agglomeration degree of capital factors. The calculation formula of the location entropy coefficient (Kclu) of the capital agglomeration level is as follows:

$$Kcluit=\frac{K_{it}}{{GDP}_{it}}/\frac{\sum K_{it}}{\sum {GDP}_{it}}$$ (4)

where *K*_*it*_ is the capital stock of city *i* in year *t*, *GDP*_*it*_ is the gross domestic product of city *i* in year *t*, and ∑*K*_*it*_ and ∑*GDP*_*it*_ are the capital stock and GDP of all cities in year *t*.

We use the perpetual inventory method to calculate each city’s capital stock, which draws lessons from Liu et al [32]. The year of the base capital stock is set as 2002, and the growth rate of fixed assets investment in the whole society is the geometric average of the growth rate from 2002 to 2017. The depreciation rate is the arithmetic average. The calculation formula is as follows

$$K_{t}=K_{t-1}(1‐\delta )+{I\prime }_{t}$$ (5)

where *I* is the investment in fixed assets of the whole society. In this paper, the average construction period of fixed assets is set at three years, which means that the amount of fixed assets added in t years is *I*′_*t*_ = (*I*_*t*_+*I*_*t*−1_+*I*_*t*−2_)/3

In the calculation of capital stock, the estimation of base capital stock plays a vital role. Different scholars show great differences in estimation methods and estimation results, which can be summarized in the following two methods. One method is to use some variables and their rates of change over a set period for the calculation. An example of this would be using the amount of investment in the base period divided by the sum of the average growth rate of investment and depreciation over a set period to calculate the base capital stock (on the premise that the growth rate of capital stock is equal to the growth rate of investment in the case of steady economic growth). The second method is to use the formula of the base capital stock calculated by Reinsdorf and Cover [33]. Assuming that depreciation begins in the second year after the completion of the investment, then the part of the t-1 year investment that is still in the capital stock at the end of the t year is I_0_ (1-δ)/(1 + g) where g is the average annual growth rate, and so on t-2 years, t-3 years. Finally, there is the initial formula of capital stock:

$$K_{0}=I_{0}\left[1+\frac{1-\delta }{1+g}+{\left(\frac{1-\delta }{1+g}\right)}^{2}+\cdots \right]=I_{0}(\frac{1-\delta }{1+g})$$ (6)

This paper also uses this formula to calculate the capital stock of the base period.

#### Control variables

After referring to the relevant literature, we select five indicators that may affect the upgrading of urban industrial structure as control variables, including the level of infrastructure construction (inf), the degree of government intervention (gov), the rare of urban economic development (pergdp), the measurement of financial scale (fin) and science and the level of investment in technology (ti). The level of infrastructure construction is measured by the per capita road area [34]. The degree of government intervention is measured by the ratio of government public budget revenue to urban GDP [35]. The level of urban economic development is measured by per capita GDP. The financial scale is measured by the ratio of the loan balance of financial institutions to the city GDP [36]. The level of science and technology investment is measured by the ratio of science and technology expenditure to urban GDP [37].

### Data sources

Based on the principle of data availability, the original data in this article come from the China Urban Statistical Yearbook and the National Statistical Yearbooks from 2008 to 2017. The missing data come from provincial statistical yearbooks and regional statistical bulletins, and the missing data of individual cities are supplemented by interpolation. Excluding the newly established prefecture-level cities and the prefecture-level cities upgraded by county-level cities from 2008 to 2017, this paper finally selects the panel data of 284 prefecture-level cities from 2008 to 2017 for analysis. The descriptive statistics of the variables are shown in Table 1.

**Table 1:** Descriptive statistics of the variables.

Variables	label	Obs	Mean	Std. Dev.	Min	Max
The rationalization of industrial structure	theil	2840	0.264	0.203	0.009	1.721
The optimization of industrial structure.	ais	2840	2.253	0.142	1.007	2.801
Capital factor agglomeration	kclu	2840	1.098	0.305	0.286	2.897
Infrastructure development level	inf	2840	11.862	8.329	0.31	108.37
Government intervention	gov	2840	0.742	0.312	0.004	0.691
Financial scale	fin	2840	1.315	0.953	0.075	15.356
Technology investment level	ti	2840	0.183	0.043	0.011	0.435
Economic development level	pergdp	2840	4.431	3.605	0.360	51.701

## Empirical test and result analysis

### Analysis of industrial structure in main years of each city

**Fig 1:**
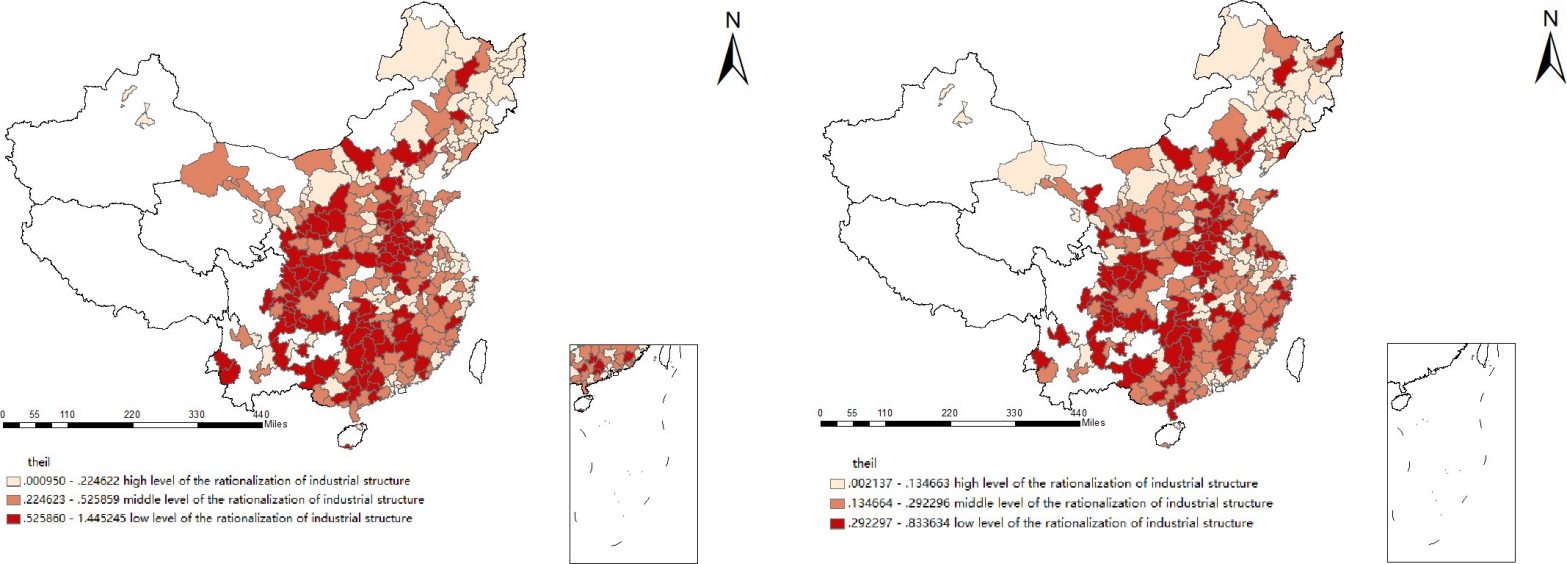
The rationalization of the industrial structure index in 2008(left), and 2017 (right). The underlying layer is quoted from the Sky Map website.

**Fig 2:**
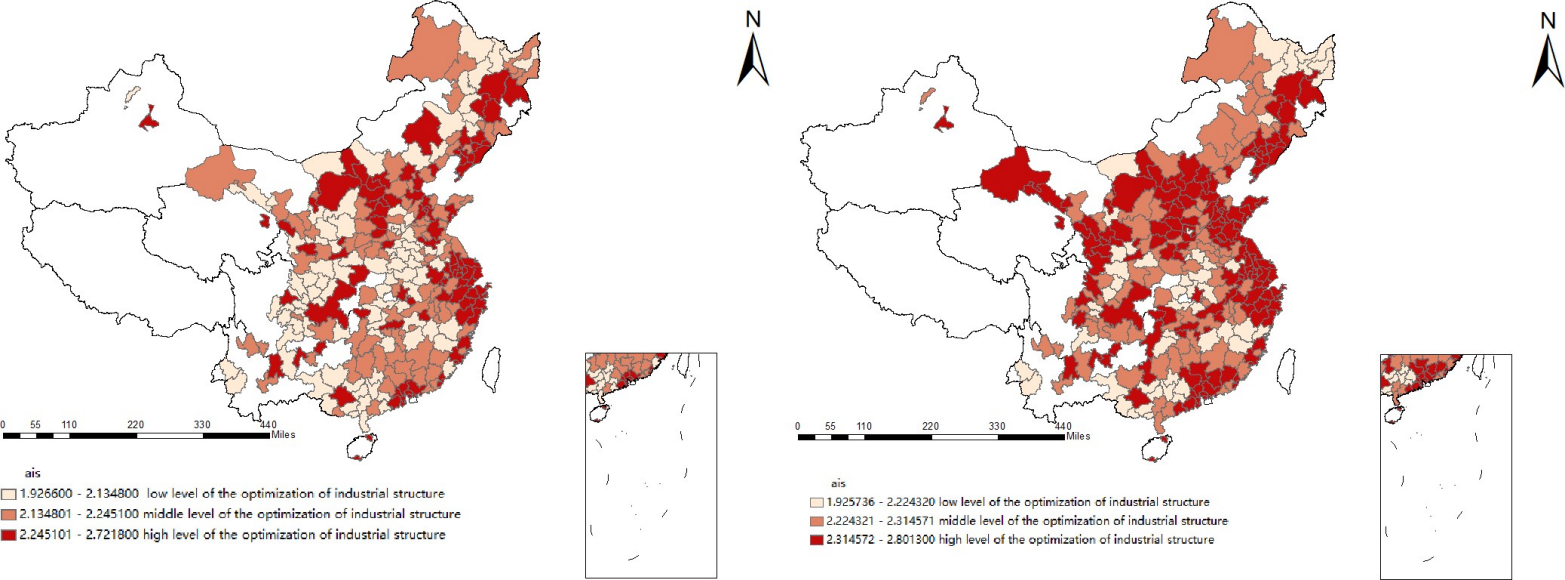
The optimization of the industrial structure index in 2008(left), and 2017 (right). The underlying layer is quoted from the Sky Map website.

As shown in Figs 1 and 2, the industrial structure upgrading index of 284 prefecture-level cities is divided into three levels. On the whole, the number of cities with medium-level and high-level industrial structures has increased significantly. In 2017, the level of rationalization and optimization of industrial structure upgrading of most cities was at the midlevel or above. From a spatial point of view, most eastern coastal cities are at a high level of industrial structure, while most central and western cities are at a low level. This also shows that the level of industrial structure of prefecture-level cities in China has obvious regional characteristics.

#### Analysis of the agglomeration of capital factors in the main years of each city

**Fig 3:**
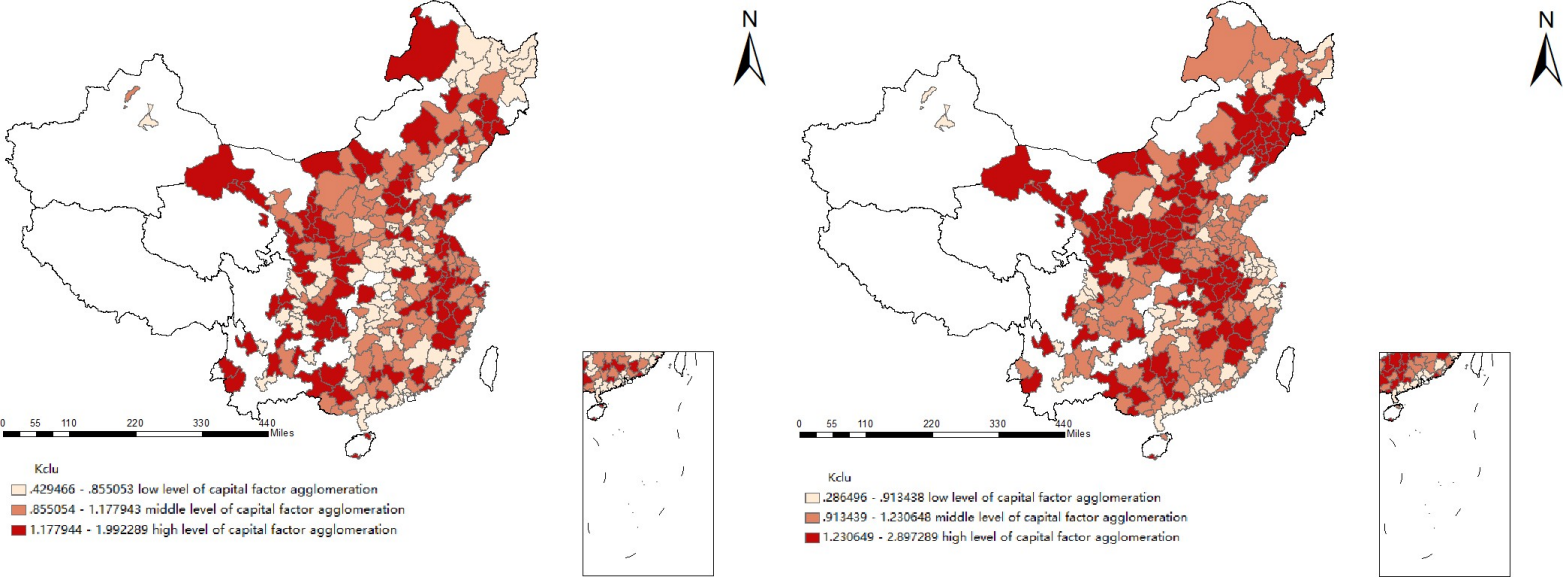
The agglomeration degree of capital factors in 2008(left), and 2017 (right). The underlying layer is quoted from the Sky Map website.

According to Fig 3, on the whole, the agglomeration degree of capital factors in 284 prefecture-level cities has been improved to a certain extent, and the proportion of cities with a high degree of capital factor agglomeration has increased significantly. From a spatial point of view, the agglomeration of capital factors has more significant regional spatial agglomeration characteristics. In 2008, the agglomeration of capital factors in the eastern coastal cities was relatively high. In 2017, capital factors tended to transfer to the central and western regions. This may be related to the central government’s policy of "large-scale development of the western region", which aims to use the surplus economic development capacity of the eastern coastal areas to improve economic and social development in the western region.

### Spatial autocorrelation analysis

#### Global spatial autocorrelation

One of the important goals of spatial econometric analysis is to test the spatial correlation of the research object, which is the prerequisite for regression analysis and use a spatial econometric model. Only by passing the spatial pattern test can the research objects with spatial correlation establish a spatial econometric model. In this paper, Moran’s I index is used to test the spatial autocorrelation of the capital factor agglomeration degree and the rationalization of the industrial structure index. The calculation formula is as follows:

$$Moran’s\;I=\frac{N\sum_{i=1}^{N}\sum_{j=1}^{N}w_{ij}(x_{i}-\bar{x})(x_{j}-\bar{x})}{\sum_{i=1}^{N}{\left(x_{i}-\bar{x}\right)}^{2}\sum_{i=1}^{N}\sum_{j=1}^{N}w_{ij}}=\frac{\sum_{i=1}^{N}\sum_{j\neq 1}^{N}w_{ij}(x_{i}-\bar{x})(x_{j}-\bar{x})}{S^{2}\sum_{i=1}^{N}\sum_{j=1}^{N}w_{ij}}$$ (7)

where *N* is the total number of regions; *w*_*ij*_ is the spatial weight of region i and region j, and the matrix composed of *w*_*ij*_ is a symmetrical matrix; *x*_*i*_ and *x*_*j*_ are the variable observations of region i and region j, respectively; the average value of the variable observations is $\bar{x}$, and the variance of the variable observation value is *S*2.

The value range of the global Moran’s I index is [– 1, 1]. If the value is greater than 0, it indicates capital factor agglomeration, or that rationalization of the urban industrial structure index is s positively and spatially correlated. A value less than 0 indicates capital factor agglomeration or the rationalization of urban industrial structure index is spatial negative correlation. The value equal to 0 indicates spatial irrelevance; that is, variables are randomly distributed in space.

Table 2 shows that Moran’s I index of capital factor agglomeration passes the significance test at the 1% level, indicating the capital factor agglomeration among Chinese cities has a very obvious spatial correlation. The rationalization of the industrial structure index also passes the significance test at the 1% level. The optimization of urban industrial structure index also passed the spatial pattern test. Due to limited space, the results are not shown.

**Table 2:** Global Moran’s I test of capital factor agglomeration and the rationalization of the industrial structure index in prefecture-level cities.

Years	The rationalization of industrial structure index			Capital factor agglomeration		
	Moran ‘s I	z-value	p value	Moran’s I	z-value	p value
2008	-0.054	-22.930	0.000	-0.012	-3.847	0.000
2009	-0.046	-19.466	0.000	-0.009	-2.267	0.012
2010	-0.048	-20.558	0.000	-0.009	-2.428	0.008
2011	-0.046	-19.417	0.000	-0.010	-2.851	0.000
2012	-0.051	-21.652	0.000	-0.010	-2.756	0.003
2013	-0.037	-15.407	0.000	-0.013	-4.313	0.000
2014	-0.039	-15.963	0.000	-0.017	-6.212	0.000
2015	-0.039	-16.028	0.000	-0.022	-8.368	0.000
2016	-0.032	-12.788	0.000	-0.025	-9.994	0.000
2017	-0.028	-11.244	0.000	-0.023	-8.964	0.000

#### Local spatial autocorrelation

**Fig 4:**
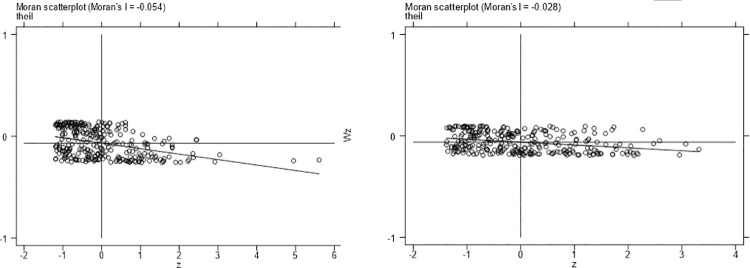
Local spatial autocorrelation test results of the industrial structure upgrading index in 2008 (left) and 2017 (right).

**Fig 5:**
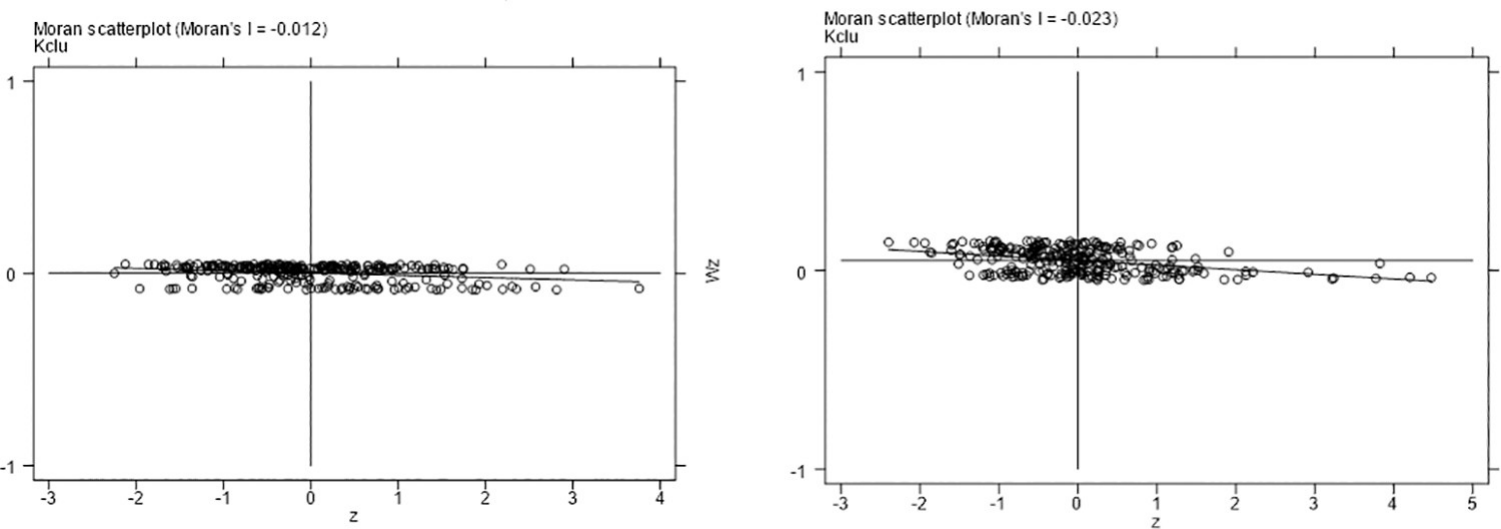
Local spatial autocorrelation test results of the level of capital factor agglomeration in 2008 (left) and 2017 (right).

Next, this paper uses the local Moran’s I index scatter diagram to test the local spatial autocorrelation of capital factor agglomeration and the rationalization of the industrial structure index. The sample is divided into four quadrants by scatter plot. When the variable is in the first and third quadrants, the variable is high-high (H-H) and low-low (L-L), respectively. When the variable is in the second and fourth quadrants, the variable is high-low (H-L) and low-high (L-H), respectively. Figs 4 and 5 show the local spatial autocorrelation test results of capital factor agglomeration and the rationalization of the industrial structure index. We find that most cities are located in the first and third quadrants. This shows that a significant positive spatial autocorrelation between the level of capital factor agglomeration and the rationalization of the industrial structure index in China. The level of capital factor agglomeration and the the optimization of the industrial structure index also have a significant positive spatial autocorrelation. Due to limited space, the results are not shown.

### Analysis of the spatial effect of capital factor agglomeration on the upgrading of urban industrial structure

The above spatial autocorrelation test shows that the urban industrial structure upgrading index and capital factor agglomeration have spatial agglomeration characteristics. Therefore, the spatial econometrics model can be used to analyze the spatial spillover effect of capital factor agglomeration on urban industrial structure upgrading. Before the spatial regression analysis, the econometric model needs to be verified. The verification results are shown in Table 3 and illustrate that at the 1% significance level, the likelihood ratio (LR) test negates the zero hypothesis that spatial Durbin model (SDM) degenerates to the spatial lag model (SAR) or spatial error model (SEM). Therefore, the SDM model is more suitable for studying the spatial relationship between the agglomeration of capital factors and the upgrading of urban industrial structure. The Hausmann test rejected the original hypothesis of random effects at 1% the level, that is, the rest of the estimates in the article were based on the fixed-effect model.

**Table 3:** The summary of LR test.

Test	LR chi2(6)	p
LR test for spatial lag	105.21	0
LR test for spatial error	104.75	0

Table 4 provides the results of SDM model with Ais and Theil as explained variables. According to the definition of the Taylor index mentioned above, the closer that the rationalization of the industrial structure is to 0, the more balanced it is. Therefore, in Table 4, the negative coefficient of Model 2 indicates the promoting effect, and the positive coefficient indicates the inhibiting effect.

**Table 4:** The estimation results of the spatial panel model.

Variable	Ais (the optimization of industrial structure) Model 1	Theil (the rationalization of industrial structure) Model 2
kclu	0.002 (0.742)	-0.042*** (0.001)
Lnpgdp	-0.026*** (0.000)	-0.012 (0.341)
Lnfin	0.025*** (0.000)	0.005 (0.708)
Lninf	0.004 (0.262)	-0.010 (0.197)
Lngov	0.013*** (0.004)	-0.023* (0.011)
Lnti	0.018*** (0.001)	0.001 (0.896)
W * kclu	0.592*** (0.000)	0.121 (0.165)
W * lnpgdp	0.014 (0.492)	-0.153*** (0.001)
W * lnfin	-0.015* (0.100)	0.093*** (0.000)
W * lninf	-0.042 (0.486)	0.197 (0.121)
W * lngov	-0.031 (0.498)	-0.283** (0.010)
W * lnti	0.018 (0.530)	0.684*** (0.000)
ρ	0.364*** (0.000)	0.436*** (0.000)
R^2^	0.5173	0.0531
Log-likelihood	4563.6993	2702.0496
Obs	2840	2840

Notes: The p values are given in the parentheses.

***, **, and * indicate statistical significance at 1%, 5%, and 10% respectively.

According to the estimated results in Table 4, the spatial spillover coefficients of the industrial structure upgrading index are 0.364 and 0.436, which are significant at the level of 1%. This is consistent with the results of the spatial autocorrelation test. This shows that the upgrading of urban industrial structure is affected not only by the internal factors of the city, but also by the spatial spillover effects of various factors in neighboring cities. The spatial spillover effect can narrow the gap between urban industrial structure levels, which is conducive to improving the urban industrial structure level overall.

Table 4 provides the regression results of the SDM model with the optimization and rationalization of urban industrial structure explained variables.

The results show that the agglomeration of capital factors can promote the optimization and rationalization of the urban industrial structure. Among them, the effect on the rationalization of industrial structure is not significant, but the impact on the rationalization of industrial structure is significant.

Specifically, according to Model 1, the influence coefficient of capital factor agglomeration on the optimization of urban industrial structure is 0.002, but it is not significant. This shows that the agglomeration of capital factors has promoted the evolution of local cities from the dominant position of the primary industry to the dominant position of the secondary and tertiary industries.

According to Model 2, the agglomeration of capital factors plays an obvious role in promoting the local urban industrial structure’s rationalization, which has passed the significance level test of 1 percentage point, and its coefficient is -0.042. This shows that every 1% increase in the concentration of capital factors in the city will increase the level of rationalization of the industrial structure of the local city by 0.042 percentage points, indicating that the agglomeration of capital factors can promote upgrading the local industrial structure and improve the degree of industrial coordination and the efficiency of resource allocation.

From the perspective of the coefficient of spatial lag, the agglomeration of capital factors promotes upgrading the industrial structure of the surrounding cities, but the spillover effect is not obvious. This may be due to the restriction of geographical distance, which makes the spatial spillover effect of factor agglomeration have regional boundaries.

Next, this paper will further analyze the spillover effects of capital factor agglomeration, and decompose the spatial spillover effects of several variables concerning the upgrading of urban industrial structure into direct effects, indirect effects and total effects by drawing lessons from the methods of Chen and Zhang [38,39]. The results of the effect decomposition are shown in Table 5.

**Table 5:** Decomposition results of spatial effects.

Variable	Ais (the optimization of industrial structure)			Theil (the rationalization of industrial structure)		
	Direct effect	Indirect effect	Total effect	Direct effect	Indirect effect	Total effect
kclu	0.003 (0.667)	0.706*** (0.000)	0.709*** (0.000)	-0.042*** (0.001)	0.081 (0.571)	0.039 (0.381)
Lnpgdp	-0.026*** (0.000)	0.010 (0.671)	-0.016 (0.516	-0.012 (0.372)	-0.071*** (0.002)	-0.084*** (0.000)
Lnfin	0.026*** (0.000)	-0.013 (0.220)	0.013 (0.146)	0.005 (0.661)	-0.044*** (0.003)	-0.039*** (0.000)
Lninf	0.004 (0.259)	-0.043 (0.548)	0.013 (0.146)	-0.010 (0.160)	0.110 (0.103)	0.100 (0.142)
Lngov	0.013*** (0.002)	-0.039 (0.477)	-0.026 (0.637)	-0.024*** (0.006)	0.150*** (0.004)	0.126** (0.015)
Lnti	0.018*** (0.000)	-0.015 (0.683)	0.003 (0.931)	-0.001 (0.993)	-0.347*** (0.000)	-0.348*** (0.000)

Notes: The p-values are given in the parentheses.

***, **, and * indicate statistical significance at 1%, 5%, and 10%, respectively.

As shown in Table 5, from the perspective of direct effect, the estimation coefficient of the capital factor agglomeration to the optimization of industrial structure is not significant. The estimation coefficient of industrial structure rationalization is -0.003, which is significant at the 1% level. This shows that the agglomeration of capital factors can optimize the efficiency of resource allocation and improve the degree of industrial coordination. Still, it does not significantly improve the optimization level of the local industrial structure.

From the perspective of indirect effects, the correlation coefficient of capital factor agglomeration to optimize the industrial structure of surrounding cities is significantly positive, and passes the test at the significance level of 1%. However its effect on the rationalization of industrial structure in the surrounding cities is not obvious. This shows that the agglomeration of capital factors can increase the proportion of secondary and tertiary industries in the surrounding cities, but cannot improve the level of industrial correlation and the efficiency of resource allocation in the surrounding cities. As a result, it weakens the positive promoting effect of capital factor agglomeration on the upgrading of industrial structure in surrounding cities. This shows that the flow mechanism of factors among cities in China is not smooth, and the spatial spillover of capital factor agglomeration is restricted by geographical distance. Local protectionism will make the spatial spillover effect of factor agglomeration have regional boundaries. Although the degree of marketization in China has obviously improved after the reform and opening up of markets, the allocation efficiency of factors among cities is not high. The market economic system still needs to be improved, thus affecting the spillover effect of capital factors on the surrounding cities.

From the overall effect, the estimation coefficient of capital factor agglomeration to the optimization of urban industrial structure is 0.709, which is significant at 1%, but the estimation coefficient of urban industrial structure rationalization is 0.039, which is not significant. On the whole, the agglomeration of capital factors can promote the upgrading of the urban industrial structure, but the quality and efficiency are not high.

Table 5 also illustrates the results of the control variables. The level of economic development promotes the rationalization of the industrial structure of the surrounding cities, but does not promote the optimization of the urban industrial structure. Thus it can be seen that the improvement of the level of urban economic development plays a significant role in upgrading the industrial structure of the surrounding cities. Cities with better economic development play a leading role in the surrounding region. The industrial division of labor and cooperation among cities can improve factor allocation efficiency and enhance the degree of industrial coordination. The financial scale has significantly promoted the rationalization of the industrial structure of the surrounding cities. A possible reason is that the development of the financial sector will facilitate the allocation of funds from less productive projects to more productive projects. It is helpful improving the economic benefit and scale expansion and then optimizing the urban industrial structure. The direct effect, indirect effect and total effect coefficient of the infrastructure construction level are not significant. The reason may be that although China’s urban infrastructure construction is in the process of continuous improvement, its operational efficiency and external effects have not been brought into full play. It has not played an obvious role in promoting the upgrading of the urban industrial structure. This may be because although China’s urban infrastructure construction is in the process of continuous improvement, its operational efficiency and external effects have not been brought into full play. The estimation coefficient of the direct effect of the degree of government intervention on the rationalization of industrial structure is significantly negative.

In contrast, the estimation coefficient of indirect effect is significantly positive. This shows that government intervention is mainly reflected in promoting the industrial upgrading of local cities, but restraining the surrounding cities. The possible reason is the prevalence of local protectionism, which may lead to competition for production resources between prefectural and municipal governments. The direct effect coefficient, indirect effect coefficient and total effect coefficient of science and technology investment are all significantly negative. This shows that with the increase of local government investment in science and technology, the city’s enterprise innovation activities have become more active. The proportion of high value-added output has increased, promoting the upgrading of the city’s industrial structure from the micro to the whole.

## Conclusions and policy implications

The agglomeration of capital factors has a spatial spillover effect, which is an important way to speed up the upgrading of the urban industrial structure and improve the resource utilization efficiency. This paper obtains data on capital factor agglomeration by calculating the capital stock of 284 prefecture-level cities in China from 2008 to 2017, while the spatial spillover effect of capital factor agglomeration on the upgrading of urban industrial structure was investigated by using the spatial econometric model under the fixed effect. The main conclusions and policy recommendations are as follows:

First, the spatial pattern of capital factor agglomeration and urban industrial structure upgrading shows the characteristics of regional agglomeration. The degree of urban industrial structure upgrading and capital factor agglomeration is increasing year by year, but there are still few high-level cities. Second, the agglomeration of capital factors can significantly promote the upgrading of local industrial structure. The economies of scale brought by agglomeration can improve the degree of industrial coordination and the efficiency of resource allocation in local cities. At the same time, the upgrading of the urban industrial structure has a significant spatial spillover effect, the improvement of the upgrading level of the local urban industrial structure is affected by the spatial spillover effect of neighboring cities, and the correlation of this effect is significantly positive. Third, the agglomeration of capital factors promotes upgrading the industrial structure of the surrounding cities, but it is not statistically significant. This shows that the factor flow mechanism among Chinese cities is not smooth, and the spatial spillover of capital factor agglomeration is restricted by geographical distance. The fact that economic gravity gradually decays with increasing geographical distance is obvious. Local protectionism will lead to a certain regional boundary of the spatial spillover effect of factor agglomeration, which affects the spillover effect of capital factors on the surrounding cities. Although the degree of marketization in China has been significantly improved after the reform and opening up of markets, the efficiency of factor allocation between cities is not high. The market economic system still needs to be improved.

According to the results of the previous discussion, to strengthen urban industrial construction and optimize the urban governance model, this paper puts forward the following policy implications. First, improves the flow mechanism of capital factors between cities. The government should breakdown the institutional barriers between regions, eliminate the institutional obstacles to the circulation of capital factors, promote the reform of market-oriented allocation of capital factors, relax capital access controls, and promote the flow of capital factors to more efficient industries. This can improve the efficiency of factor allocation, and improve the degree of urban industrial coordination and promote the upgrading of industrial structure.

Second, due to the spatial spillover effect of upgrading the industrial structure between cities, the central government needs to plan and establish urban agglomeration according to the actual situation to achieve the coordinated development of urban industries. At the same time, the local government should fully consider the factor endowment, development level and industrial structure of different regions, guide the flow of capital factors through relevant policies and regulations, enhance the agglomeration level of capital factors, adopt complementary strategies in the region, actively seek regional cooperation between cities, and further realize the coordinated development of industries between prefecture-level cities.

Finally, a multilevel capital market system should be established. At present, most areas are still dominated by government investment, and it is necessary to improve the financing environment and broaden financing channels. At the same time, the government should actively develop private banks and foreign-funded financial institutions, introduce private capital and all kinds of investment funds, and solve the problems of high financing costs and financing difficulties of enterprises. At present, most of the capital factors are highly concentrated in cities in eastern China, which can lead to inefficient allocation of financial resources makes it is difficult to maximize the impact of capital factor agglomeration on the upgrading of industrial structure. The central government should strengthen the financial cooperation between the eastern and western regions, actively guide the flow of capital factors from the eastern regions to the central and western regions, enhance the diffusion and radiation effects of capital factors, and give full play to the economies of scale and resource allocation effects of financial agglomeration to promote the agglomeration of capital factors to serve the upgrading of urban industrial structure better.

The limitation of this study is that the calculation of the two indicators of capital factor agglomeration and urban industrial structure upgrading is not comprehensive. It is necessary further to construct a more comprehensive evaluation index system of them. For example, capital factors can be divided into government investment, private investment and venture capital. We can construct the index evaluation system according to this classification. At the same time, urban green development can be integrated into the evaluation index system of industrial structure upgrading. These are all directions that can be further studied.
